# Influence of Different Types of Root Canal Irrigation Regimen on Resin-based Sealer Penetration and Pushout Bond Strength

**DOI:** 10.7759/cureus.7807

**Published:** 2020-04-24

**Authors:** Chundangaparambil Pushpahasan Sreedev, Iswarya Raju, Karthick Kumaravadivel, Sebeena Mathew, Boopathi Thangavel, Deepa Natesan Thangaraj

**Affiliations:** 1 Conservative Dentistry and Endodontics, MES Dental College, Malappuram, IND; 2 Conservative Dentistry and Endodontics, KSR Institute of Dental Science and Research, Tiruchengode, IND

**Keywords:** citric acid, diode laser, edta, endodontic irrigants, root canal sealers

## Abstract

Introduction

The main objective of root canal treatment is to eliminate the micro-organism from the root canal system and three-dimensional obturation. The proper cleaning and shaping can be accomplished only by using appropriate instruments and effective irrigants during the root canal treatment.

Aim

To evaluate the influence of three different final irrigation regimen on depth of penetration of root canal sealers and push-out bond strength of obturation material.

Materials and methods

Thirty-six extracted single-rooted mandibular premolar human teeth with straight canals were decoronated and instrumented according to groups. Group I: Root canals were irrigated with 3% sodium hypochlorite (NaOCl), then irradiated with 980 nm diode laser (n = 12), Group II: Root canals were irrigated with 3% NaOCl, followed by 17% ethylenediaminetetraacetic acid (EDTA) (n = 12), Group III: Root canals were irrigated with 3% NaOCl, followed by 10% citric acid (n = 12). In each sample, single cone obturation was done with gutta-percha using AH plus sealer incorporated with rhodamine B dye. After seven days coronal, middle and apical thin cross sections were made for evaluation of dentinal tubule sealer penetration depth and pushout bond strength using confocal laser scanning microscope (CLSM) and universal testing machine, respectively. Statistical analysis among the three groups was done by using Kruskal-Wallis and post hoc test.

Results

Mean tubular penetration depth between diode laser (136.57 ± 48 µm), EDTA (130.56 ± 53 µm) and citric acid (113.37 ± 34 µm; P < 0.05) showed statistically highly significant results. Pushout bond strength did not differ significantly between diode laser (1.21 ± 0.48 Mpa), EDTA (1.05 ± 0.45 Mpa) and citric acid (0.93 ± 0.44 Mpa; P > 0.05).

Conclusion

Mean tubular penetration depth of AH plus sealer was better in diode laser than in EDTA and citric acid. Average push-out bond strength of obturation material did not differ significantly between diode laser, EDTA and citric acid.

## Introduction

The purposes of root canal treatments are to eliminate microorganisms from the root canal system and to prevent re-contamination. Because of the complex anatomy of the root canal system, it is impossible to achieve complete disinfection of the root canal using instrumentation alone. Irrigation is an important compliment to instrumentation because it removes or wash out bacteria, debris, and necrotic tissue present inside the canal [1].

The smear layer consists of organic and inorganic components, such as vital or necrotic pulp tissue, microorganisms, salivary components, blood cells, and dentinal debris. It has an amorphous and irregular appearance and consists of two separate layers [2]. The removal of the smear layer is still a matter of debate. In contrast, some experts believe that the smear layer should be removed from the surface of the canal wall because it can not only harbour bacteria but also reduce dentin permeability by preventing penetration of root canal sealers into the dentinal tubules. Thus it can act as a barrier between the obturating materials and root canal wall that may interfere with the formation of an appropriate seal [3-6].

Sodium hypochlorite (NaOCl) is the most common irrigant used during root canal therapy. But it has weak action towards the removal of inorganic part of the smear layer.

Chelating agents, such as ethylenediaminetetraacetic acid (EDTA), citric acid (CA), maleic acid (MA), phosphoric acid, and combinations of EDTA and NaOCl have been used to remove the smear layer [7].

Diode lasers emitting at 980 nm have better research and clinical applications because they transmit energy through thin flexible fibers that are compatible with the morphology and curved shapes of root canals. Diode lasers have a good penetration potential, with high absorption peaks for melanin and hemoglobin and limited interactions with water and hydroxyapatite [8]. Its power output ranges from 0.5 to 7 W and can be used with different operating modes like continuous wave, pulsed power and chopped mode [9]. Wang et al. demonstrated the effect of 980-nm wavelength diode laser on removal of smear layer and debris and degree of apical leakage in obturated teeth [10]. Certain studies found that citric acid of variable concentrations cleansed the canal walls and left the dentinal tubules open, which allowed intimate adaptation of resin-based materials. This may help in more adaptation of epoxy resin-based sealers to root canal walls [11].

Sealer penetration into the dentinal tubules is a desirable property, because it would entomb residual debris and microorganisms and it can maintain them far from nutrient sources. Moreover, deep endodontic sealer penetration is notably important because it decreases the interface between gutta-percha and root dentin and it may improve the retention of the filling mass by mechanical locking [12].

Bond strength testing is the best measure of adhesion. Adhesion of the root canal filling to the dentinal walls is advantageous in eliminating any space between the filling and the canal wall and also resists dislodgement of the filling during subsequent manipulation [13].

Several studies have investigated the degree and adaptation of root canal sealers to root dentin [3-6]. However, limited studies have investigated the influence of final irrigation regimen on depth of penetration of root canal sealers and push out bond strength of obturation material.

The aims of the present study were to compare the effect of diode laser, 17% EDTA and 10% citric acid on penetrability of a resin-based sealer into dentinal tubules using a confocal laser scanning microscope (CLSM) and push-out bond strength of gutta-percha. The null hypotheses tested were that the different irrigation regimen has no difference in sealer penetrability and push-out bond strength.

## Materials and methods

Tooth preparation

The present in vitro study was approved by the Ethical Committee from the KSR Institute of Dental Science and Research (Ref: 079/KSRIDSR/EC/2014). The study was conducted in the Department of Conservative Dentistry and Endodontics, KSR Institute of Dental Science and Research, Thiruchengode, Tamil Nadu. Sample size was calculated using online sample size calculator (Raosoft.Inc.). The power of the study was set at 80%. Thirty-six extracted non-carious single-rooted mandibular premolar human teeth with straight canals were used in this study. Before the experiment, root surfaces of all specimens were cleaned mechanically and ultrasonically. The crowns of the teeth were decoronated at the cemento-enamel junction with a high-speed diamond wafering blade (Struers Ltd, Mumbai) having 300 µm-thickness and 160 mm diameter under water irrigation. The canals were cleaned and instrumented by the crown-down technique up to ProTaper F3 (Dentsply Maillefer, Ballaigues, Switzerland). The working length was visually determined 1 mm shorter from the anatomical apical foramen. Total number of samples were randomly divided into three groups (n = 12) based on the irrigation regimen.

Group I: Root canals were irrigated with 3% NaOCl (Neelkanth Healthcare, Boranada, India) for 2 min, then irradiated with 980 nm diode laser (SIROlaser 2.2; SIRONA Dental, Bensheim, Germany) in two cycles of 7 seconds with 5 W power in the continuous mode in a whirling motion from apical to coronal portion of root canal using a 200-μm diameter flexible optical fiber. Sodium hypochlorite irrigation was done in between and after laser irradiation. Group II: Root canals were irrigated with 3% NaOCl for 2 min, followed by 17% EDTA (Fisher Scientific, Mumbai, India) for 1 min. Group III: Root canals were irrigated with 3% NaOCl for 2 min, followed by 10% citric acid (Freshly prepared) for 1 min. Final irrigation and in between two active irrigants of all groups were done with 5 ml of normal saline to terminate any solvent action of the irrigants and to remove any precipitates that may have formed from the irrigants. The root canals were dried with paper points. The teeth were then randomly divided into three groups of 12 teeth each.

Equal parts of paste A and paste B of an epoxy resin-based sealer (AH Plus, Dentsply Maillefer) were mixed with 0.1% rhodamine B (RITC; Sigma-Aldrich, St. Louis, MO). The endodontic sealer was applied in the canal 1-mm shorter than the WL using ProTaper F3 gutta-percha cone (Dentsply Maillefer) and single cone obturation was done. The specimens were stored at 37°C and 100% humidity for seven days to allow the sealer to set. A 1-mm thick horizontal slice was prepared from coronal middle and apical portion of the root using a diamond disc (Diamond Disc Superflex 910S/220, Italy) under water cooling and apical 3 mm was discarded. The coronal surfaces of the slices were polished with Arotec paste (Arotec, Cotia, SP, Brazil) to eliminate dentin debris generated during the cutting procedures and to produce a highly reflective surface.

Sealer penetration

**Figure 1:**
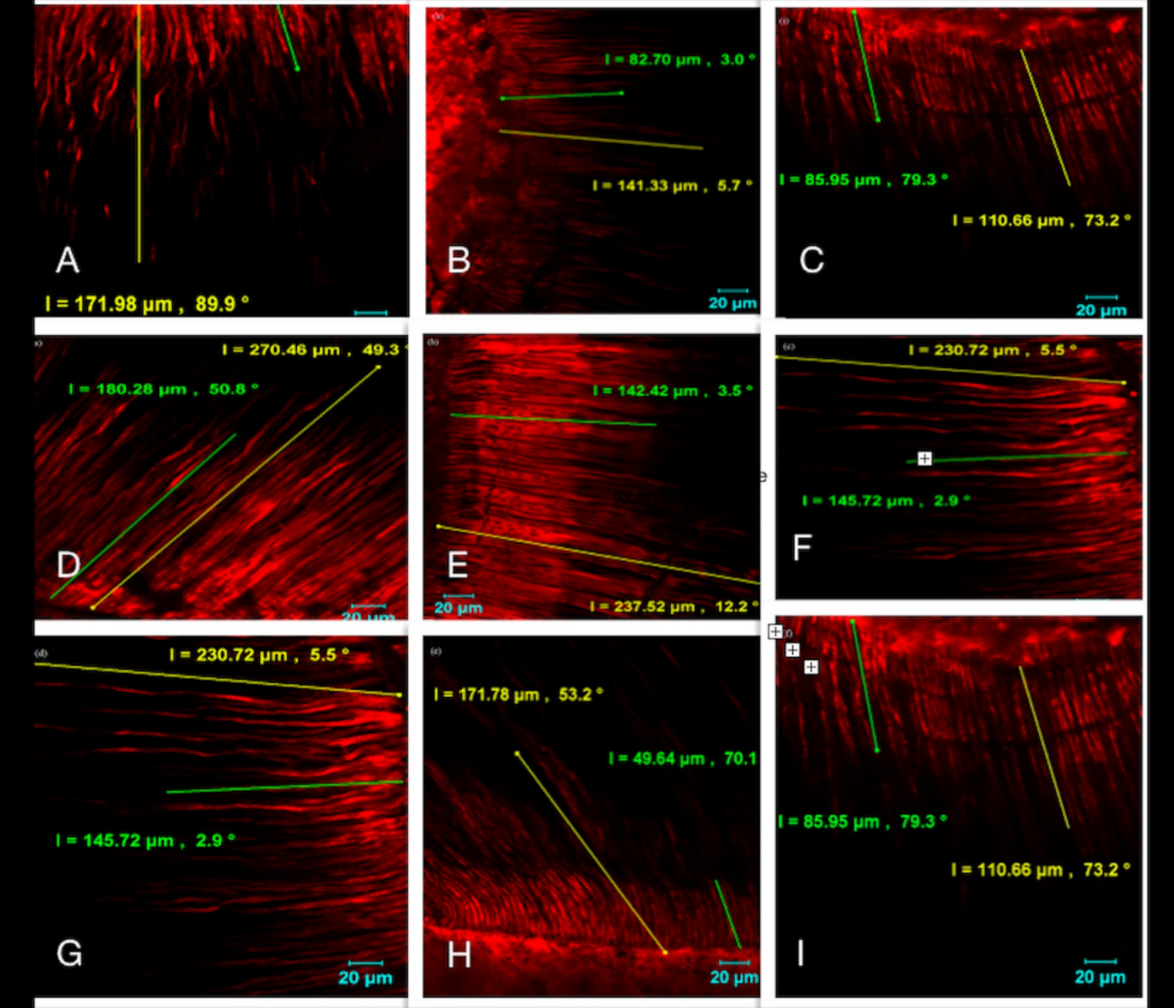
Confocal microscopic image showing the penetration of different groups. (A) Citric acid coronal, (B) Citric acid middle, (C) Citric acid apical, (D) Diode coronal, (E) Diode middle, (F) Diode apical, (G) EDTA coronal, (H) EDTA middle, (I) EDTA apical. EDTA: Ethylenediaminetetraacetic acid

All the slices were examined using an Olympus Fluoview 1000 confocal laser scanning microscope (CLSM) (Olympus Corporation, Tokyo, Japan) with 40× magnification. The image settings were 70-μm depth with 800 × 800 pixels. Four images from different area of each section were taken and maximum penetration depth of the sealer in each image was measured in micrometers. Mean depth for each section is calculated. Images were captured with the HCX PL APO 40/1.25-0.75 oil lens (Leica), and acquired with the IM50 Image Manager Software, v1.20 (Leica Microsystems, Buffalo Grove, IL, USA) (Figure 1). When penetration was observed, the length of the sealer tag from the canal wall along the entire tubule was recorded in microns. The canal wall served as the starting point, and sealer penetration into dentinal tubules (sealer tags) was calculated to a maximum depth of 1000 μm with the ruler tool of the Leica Application Suite Advanced Fluorescence Lite software (Leica Microsystems).

Push-out bond strength

After the sealer penetration examination, the root slices were prepared for push-out bond strength test. The thickness of the root slices was measured using a vernier caliper, the filling material was then loaded with a 0.5-mm diameter cylindrical stainless steel plunger. Loading was applied on a universal testing machine (Z050, Zwick/Roell, Ulm, Germany) at a speed of 0.5 mm/min in an apical coronal direction to avoid any interference because of the root canal taper during push-out testing. The bond strength was determined using a computer software program connected to the universal testing machine.

The maximum load applied to the filling material before debonding was recorded in Newtons.

The load at failure recorded in Newtons (N) was divided by the interfacial area to express the bond strength in megapascals (MPa).

Push-out bond strength (MPa) = Maximum load (N) / Adhesion area of root canal filling (mm^2^). The ‘bonded (adhesion) area’ of each slice was calculated using the formula below: 2πrh, where ‘π’ is equal to 3.14, ‘r’ is the radius of root canal filling and ‘h’ is the thickness of the slice. Coronal and apical radius of filling material assumed to be the same (1 mm). After the bond strength test was performed, both sides of the root slices were examined under a light microscope at 25× magnification to determine the failure mode. Modes of bond failure were considered as follows: (1) adhesive; at filling material-dentin interface, (2) cohesive; within filling material, and (3) mixed failure.

## Results

Both push-out bond strength and tubule penetration data were statistically analyzed by using Kruskal-Wallis and post hoc test (STATISTICA 7.0, StatSoft Inc., Tulsa, OK, USA). The level of significance was set at p < 0.05. Table 1 shows the statistical result of depth of dentinal tubular sealer penetration in the test groups. Diode laser (136.57 ± 48 µm) group showed the highest sealer penetration into dentinal tubules than EDTA group (130.56 ± 53 µm) and citric acid group (113.37 ± 34 µm) (p < 0.05). Coronal third showed higher depth of sealer penetration and apical third with lower depth of sealer penetration into dentinal tubules (Figure 1).

**Table 1:** Dentinal tubule penetration depth (µm) in various test groups (p < 0.05).

Root canal third	Root canal filling materials		
	Diode laser	EDTA	Citric acid
Coronal	185.16 ± 43.43	179.07 ± 56.43	139.02 ± 35.30
Middle	131.30 ± 22.02	126.48 ± 23.68	117.54 ± 15.32
Apical	93.25 ± 21.22	86.14 ± 20.05	83.53 ± 19.57
Median	129.35	120.45	110.00

No significant difference was found amongst the bond strength values of the test groups. Citric acid group (0.93 ± 0.44 MPa) showed lower bond strength values than diode laser (1.21 ± 0.48 MPa) and EDTA group (1.05 ± 0.45 MPa). Diode laser group showed higher bond strength. Comparison of bond strength in root canal thirds showed coronal third with higher bond strength and apical third with lower bond strength. Table 2 shows the statistical result of push-out bond strength of the test groups. Stereomicroscopic failure analysis is illustrated in Table 3. The predominant failure mode was mixed failure in diode laser and EDTA group whereas in citric acid group more adhesive failures were found.

**Table 2:** Bond strength mean values and standard deviations (in MPa) for filling material displacement from intraradicular dentine in each third using the push-out test in specimens (p < 0.05).

Root canal third	Root canal filling materials		
	Diode laser	EDTA	Citric acid
Coronal	1.41 ± 0.51	1.22 ± 0.46	1.10 ± 0.42
Middle	1.22 ± 0.46	1.02 ± 0.47	0.89 ± 0.45
Apical	1.02 ± 0.42	0.92 ± 0.42	0.80 ± 0.42
Median	1.21	1	0.99

**Table 3:** Stereo microscopic evaluation of failure mode after push-out bond strength. CT: Cervical third; MT: Middle third; AT: Apical third.

	Diode laser			EDTA			Citric acid		
Failure mode	CT	MT	AT	CT	MT	AT	CT	MT	AT
Adhesive	30	20	20	30	30	40	40	30	30
Cohesive	30	30	20	40	30	20	10	30	20
Mixed	40	50	60	30	40	40	50	40	50

## Discussion

The presence of a smear layer on the canal walls can reduce dentin permeability and may hinder penetration of sealers into dentinal tubules [14]. Therefore, to improve the quality of the root canal filling its removal is essential, as the three different root canal irrigation regimens affect the bond strength and sealer penetration variably on the same epoxy resin-based sealer. The null hypothesis that there is no difference among the groups tested was rejected. The integrity of sealer penetration using single master cone technique is comparable to that of cold lateral compaction, and Thermafil technique [15]. Sealer penetration into the dentinal tubules could improve sealing of a root filling by increasing the surface contact area between the root filling materials and dentinal walls [16]. Furthermore, retention of root filling material might be improved by mechanical locking.

CLSM was used for evaluation of the dentinal tubule penetrations instead of scanning electron microscope because it helps to create standard and reproducible three-dimensional imaging of the samples without damaging them [17]. It also allows evaluation of tubule penetration and adaptation of sealer quickly and objectively in a panoramic image manner obtained at a lower magnification through the fluorescent effect of rhodamine B. It has been previously shown that rhodamine B is a safe dye that has no effect on the setting of sealers [18].

The results of this study showed that the maximum penetration of all the resin sealers was seen at the coronal third, followed by the middle third and least in the apical third. Various authors have demonstrated regional variation in the depth of tubular penetration. The apical dentin displays less tubule density, with some areas completely devoid of tubules [19]. According to some experts, it is harder to remove the smear layer from the apical third than from the other thirds. At this third, the delivery of the irrigant is minimized and dentin showed less tubule density or even areas with absent of tubules [20]. Modified organic matrix layer with an amorphous form and tubule visibility were seen at low power whereas sparse lava-like formations with opened tubules were seen at higher power. These altered root canal micro changes could be dissolved by NaOCl which may facilitate the better sealer penetration [21].

Irradiation of Nd:YAG laser in root canals negatively influenced the adaptation of hydrophilic resin-based sealers to the dentin walls, and sealer penetration into the dentinal tubules. The wavelengths of these lasers caused the melting and resolidification of dentin, which might have led to occlusion of dentinal tubules and it was the reason for impaired permeability of canal walls [22]. However, in the present study diode lasers caused ablation of dentin and thus exposing the dentinal tubules. This phenomenon causes an increase in dentin permeability. These lasers also promoted the loss of dentin hydration and thus improving the sealing capacity of this sealer. Root canal walls also exhibited modified smear layer and some cracks. The choice for the power setting and duration used in the present study was based on the results of Wang et al., who demonstrated that these parameters yielded a temperature rise of almost 8.1°C, which does not exceed the threshold supported by the periapical tissues without causing thermal damage [10,23].

It is difficult to completely remove the residual smear layer to allow sealer penetration, particularly in the apical third of the root, because the smaller size of the apical third compared with the other thirds impedes the circulation and impaired action of the irrigating solutions. In addition, there are fewer dentinal tubules due to increased tubular sclerosis. Acoustic and hydrodynamic properties of irrigants also might have improved the effectiveness of irrigating solutions in the apical region [18]; agitation with a laser has been used in endodontic therapy to reduce the number of bacteria and to modify the surface of the root canal [24]. Surface tension and its viscosity are two of the most essential parameters related to fluid flow. Wettability plays a major role for obtaining better contact time of irrigants in root canal walls. Surface tension is a phenomenon of intramolecular attraction, and this can be accomplished by the addition of surfactant or raising temperature [25]. Few studies showed the improved action of endodontic irrigants by raising temperatures and adding surfactants. However, such alterations were not considered in the present study [26].

Photon-induced photoacoustic streaming (PIPS) is a laser-activated irrigation technique that cannot completely remove bacteria from infected root canal dentinal tubules but can generate less infection and removes biofilm better than passive ultrasonic irrigation technique [27]. Advanced noninvasive light-activated disinfection (ANILAD) is a more efficient type of light-activated disinfection that shows the better penetration into dentinal tubules and the bacterial kill rate [28].

The push-out test has been used to evaluate the dentin bond strength of root canal filling materials. This method allows the standardization of the specimens and evaluation of very low bond strength values. For allowing the assessment of bond strength at different root levels, we obtained root horizontal sections from each third of the roots and plunger kept to a tip size of 0.5 mm to prevent the touching the dentin walls during the test. It has also previously been shown that the location of the slice might not have a significant effect on bond strength.

Some studies used only the sealer for filling the root canal to eliminate the influence of the two interfaces in the push-out test. It could eliminate the influence of gutta-percha in adhesion test. Also as a cylindrical plunger was used, the irregular shape of root canals can influence the results [24,29].

When smear layer was removed, the highest adhesive force values associated with epoxy resin-based sealers occurred with a final flush of NaOCl. Epoxy resin-based AH Plus showed better penetration into microirregularities due to its creep capacity and long setting time, which might increase the mechanical interlocking between sealer and root dentine. In addition, cohesion amongst sealer molecules, which increases its resistance to removal and/or displacement from dentine, contributes to the greater bond strength [30].

## Conclusions

Within the limitations, this in vitro study concluded that the diode laser is useful for better root canal sealer penetration and provides higher push-out bond strength than EDTA, citric acid and can be considered for the clinical root canal treatment procedures. The maximum sealer penetration was achieved in coronal third. The sealer penetration was least in the apical third area.
